# Disaster differentially affects underserved communities

**DOI:** 10.1016/j.jacig.2022.04.004

**Published:** 2022-04-30

**Authors:** Margaret P. Huntwork, Thao P. Le, Keith L. Winfrey, John C. Carlson

**Affiliations:** a Tulane University School of Medicine, New Orleans, La; b NOELA Community Health Center, New Orleans, La

## Abstract

Patients with barriers to care, including poverty and language barriers, often live in lower-cost, disaster-prone areas. Partnering with community clinics enables allergists to reach underserved patients.

## Case report

A 70-year-old woman has a 20-year history of nonallergic asthma without eosinophilia. Symptoms include chest tightness, wheeze, cough, shortness of breath, and frequent nocturnal awakenings due to dyspnea. Triggers include weather changes. Asthma is currently controlled using budesonide-formoterol. She had 1 emergency department visit for asthma in the previous 12 months and was discharged with a 5-day course of prednisone.

The patient is predominantly Vietnamese-speaking with limited English proficiency and resides in Eastern New Orleans, an area of the city disproportionately affected by disasters. She lives with her spouse, who also suffers from chronic health issues. Her relatives moved to Eastern New Orleans as refugees of the war in Vietnam, and relatives sponsored her resettlement near them 20 years ago. She receives health care at the New Orleans East, Louisiana (NOELA) Community Health Center near her home, where she is cared for by a local Internal Medicine physician, a Vietnamese-speaking physician assistant, and the allergy team from Tulane University. Her local pharmacist speaks Vietnamese and creates medication labels for her in Vietnamese. Through use of a medical interpreter, the patient provided informed consent for us to share her history and experiences.

In August 2005, the patient did not evacuate from Eastern New Orleans preceding Hurricane Katrina. She was subsequently evacuated from the flooded city to Houston where she spent 4 months in a hotel. Her asthma became uncontrolled because she could not rely on her usual pharmacist and was unsure where to get medications. The Atlantic hurricane season (June through November) now causes her significant anxiety. She can drive but does not like to drive anywhere other than the grocery store, the pharmacy, and the clinic. If she needs to evacuate again, she is not sure where she would go, or how she would get there. She eventually regained asthma control, and now refills her medications promptly during hurricane season, but if her pharmacy or clinic ever experiences damage, she is not sure who she would call or where she would go if she ran out of medications. She feels comfortable only with her pharmacist because they share the same language, but she is not comfortable contacting a new pharmacy due to possible language barriers. She also worries about having enough food in the event she loses power due to a hurricane.

## Discussion

**Fig 1:**
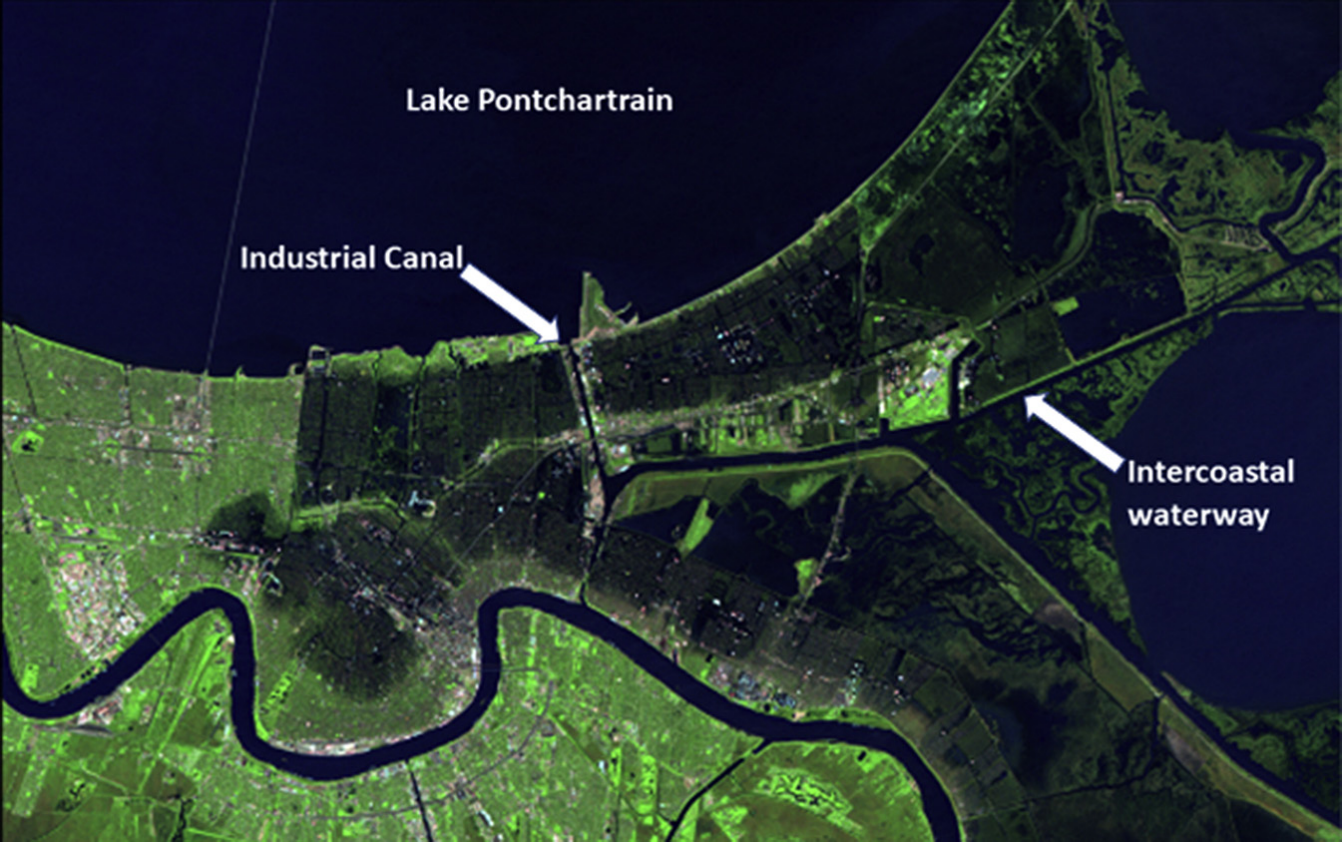
Landsat 5 satellite image of New Orleans from September 7, 2005. Darker areas are floodwaters. Eastern New Orleans is the area bounded by an industrial canal to the west, Intercoastal Waterway to the south, Lake Pontchartrain to the north, and open water to the east.

The original land developers in Eastern New Orleans aspired to convert wetlands into suburban neighborhoods for middle- and upper-class whites on the far side of an industrial canal that separates the area from the rest of the city^1^ (Fig 1). An economic crash in the 1980s left developers bankrupt. Subsequent developers built low-cost communities with limited regulations, resulting in flood-prone neighborhoods. This has worsened the spatial concentration of poverty within New Orleans.^2^ By the 2000 census, Eastern New Orleans was predominantly Black, with subsequent increases in Hispanic and Vietnamese immigrant families.^1^^,^^3^ The Vietnamese population in New Orleans began with resettlement of refugees from the war in Vietnam, growing to 12,000 by 2000.^3^

On August 29, 2005, Hurricane Katrina pushed storm surge into an inadequate levee system; the breaching of those levees left 80% of the city submerged. The flooding affected the most impoverished neighborhoods, predominantly composed of non-White individuals, especially immigrants.^2^, ^3^, ^4^ Black residents and poor residents were less likely to return because of uninhabitable homes, lack of employment, and disamenities after flooding.^2^^,^^3^ One year after the disaster, only two-third of Vietnamese participants in an earlier study had returned.^3^

Patients with chronic medical conditions delayed returning because medical infrastructure was destroyed. For example, both hospitals in Eastern New Orleans closed, replaced by 1 in 2014, 9 years after flooding. A year after the storm, 40% of working-age Vietnamese-Americans still had difficulty obtaining medications.^2^^,^^5^ They were also less likely to see a provider for their annual visit, which is instrumental in preventing development of new disease or progression of a chronic one.^5^ As Spanish-speaking and Vietnamese-speaking communities have reestablished, communities have drawn in residents who are poorer and have more medical conditions. The characteristics that led to marginalized communities settling in Eastern New Orleans remain, including propensity for flooding, limited economic opportunities, and geopolitical separation from the rest of the city.

Immigrants are especially vulnerable after disasters.^4^ According to the US Census Bureau in 2000, 93% of Vietnamese-Americans speak Vietnamese at home and 65% do not speak English very well.^3^ Language barriers during disaster recovery impair dissemination of information, make services inaccessible, and hamper financial recovery.^3^^,^^4^^,^^6^ Immigrants often have a strong cultural identity and rely heavily on their social network. Sudden displacement of support systems becomes a significant stressor, and can make recovery after a disaster more difficult.^4^^,^^6^ For many, Katrina evacuation retriggered memories of fleeing Vietnam 30 years earlier, with emotional distress and financial strain being a risk factor for the development of posttraumatic stress syndrome.^6^^,^^7^

The NOELA Community Health Center maintains a list of patients at high risk in the event of a disaster; most of the clinic’s high-risk patients are Vietnamese- or Spanish-speaking individuals. When clinic staff members contact patients, they ask about options to evacuate, need for medication, and access to food in case of persistent power outages. Clinic staff arrange refills of medications and connect patients to food banks, as needed. The clinic estimates that it reaches out to 150 patients for each hurricane that threatens the region. This was repeated for 5 hurricanes last season. Beyond the 2005 flooding, Eastern New Orleans was affected by job loss from the BP oil spill in 2010 and an EF3 tornado in 2017. In the rebuilding of medical infrastructure following the 2005 flooding, a number of federally qualified health centers have been established to care for patients within their communities, a model that substantially differs from the centralized, hospital-based care provided to patients before the flooding.^8^ Our partnership with the NOELA Community Health Center allows faculty and trainees to bolster services to patients in this disaster-prone area by providing evidence-based, subspecialty care in the setting of the Community Health Center. In return, faculty and trainees gain a deeper understanding of the intersection of medicine with history, geopolitics, health disparities, and natural disasters. Developing partnerships within underserved communities strengthens allergists as well as the patients for whom they care.^9^

## Conclusions

Patients with barriers to care, including poverty and language barriers, often live in lower-cost, disaster-prone areas. Partnering with community clinics enables allergists to reach underserved patients.
